# Intraoperative Transvaginal Ultrasonographic Evaluation for Placenta Accreta Spectrum in Placenta Previa: A Retrospective Observational Study

**DOI:** 10.1111/jog.70353

**Published:** 2026-06-04

**Authors:** Tomohiro Mitoma, Shujiro Sakata, Ayano Suemori, Hikaru Ooba, Kei Hayata, Jota Maki

**Affiliations:** ^1^ Center for Innovative Clinical Medicine Okayama University Okayama Japan; ^2^ Department of Obstetrics and Gynecology Chugoku Central Hospital Hiroshima Japan; ^3^ Department of Obstetrics and Gynecology Okayama University Graduate School of Medicine, Dentistry and Pharmaceutical Sciences Okayama Japan; ^4^ Department of Obstetrics and Gynecology Fukuyama Medical Center Hiroshima Japan; ^5^ Department of Obstetrics and Gynecology Fukuyama City Hospital Hiroshima Japan; ^6^ Fujihara Ladies Clinic, Asaminami‐ku Hiroshima Japan

**Keywords:** cervical hypervascularity, cesarean section, hysterectomy, intraoperative ultrasonography, multidisciplinary team, placenta accreta spectrum, placenta previa, transvaginal ultrasonography

## Abstract

**Aim:**

To assess the utility of intraoperative transvaginal ultrasonography (TVUS) in evaluating the duration of blood flow in cervical hypervascularity as a real time diagnostic marker for placenta accreta spectrum (PAS) in placenta previa.

**Methods:**

This single‐center historical cohort study included patients with placenta previa with intraoperative TVUS. The primary outcome was blood flow persistence time, defined as the interval from fetal delivery to disappearance of low‐velocity (≤ 2.0 cm/s) color Doppler signals in cervical hypervascularity. Two obstetricians independently reviewed surgical video recordings. Receiver operating characteristic curve analysis was performed to evaluate the diagnostic performance of flow persistence time for predicting PAS. The AUC was calculated to assess discrimination. Optimal cutoffs were based on sensitivity and specificity. Independent predictors of PAS were identified by multivariate regression.

**Results:**

PAS was diagnosed in 20 cases (38.5%). Flow persistence time was significantly longer in PAS cases (20.20 ± 14.31 min) than in non‐PAS cases (5.69 ± 2.46 min). A cut‐off of 7 min achieved 100% sensitivity and 72% specificity (AUC = 0.94), reliably excluding PAS when flow ceased within this period. A threshold of ≥ 14 min yielded 100% positive predictive value for PAS and 96% specificity for predicting hysterectomy. Flow duration remained an independent predictor in multivariable analysis (AUC = 0.95).

**Conclusions:**

Intraoperative assessment of cervical hypervascularity blood flow duration using TVUS may serve as a supportive indicator when PAS is suspected. A flow duration of < 7 min may help rule out PAS, while ≥ 14 min may suggest its presence and guide safer intraoperative management.

**Abbreviations**
AUC: area under the curve
FIGO: International Federation of Gynecology and Obstetrics
MRI: magnetic resonance imaging
NPV: negative predictive value
PAS: placenta accreta spectrum
PPV: positive predictive value
ROC: receiver operating characteristic
TVUS: transvaginal ultrasonography

## 1 Background

Accurate risk assessment of placenta accreta spectrum (PAS) in cases of placenta previa is critical for safe surgical management. Although prenatal diagnosis of PAS has improved with advances in imaging technologies, no standardized method currently exists for intraoperative assessment. In particular, there is limited evidence regarding real‐time intraoperative evaluation of placental blood flow using ultrasonography.

Previous studies have primarily focused on enhancing preoperative prediction through detailed imaging [1, 2, 3]. The introduction of magnetic resonance imaging (MRI) has enabled a more comprehensive assessment, including quantification of cervical hypervascularity [4, 5]. Practical and reproducible ultrasonography‐based scoring systems with favorable sensitivity and specificity have also demonstrated increasing clinical utility [6, 7, 8]. Preoperative planning requires the integration of imaging findings with clinical risk factors to predict PAS and the risk of massive hemorrhage, facilitating preparation for severe complications [9, 10]. Such preparation includes establishing perioperative strategies involving massive transfusion protocols, interventional radiology, and multidisciplinary specialist teams [11]. However, when unexpected placental adherence is encountered intraoperatively, forceful manual removal or delays in achieving hemostasis can lead to serious complications. Inadequate preparation may result in delayed management and worsened maternal outcomes [12]. Moreover, intraoperative decision‐making regarding PAS management, including the consideration of hysterectomy, requires experienced operators and infrastructure to support timely clinical judgment [13, 14]. In patients with placenta previa, careful assessment of abnormal placental attachment is essential, and surgical approaches must be guided by the risk of PAS [10]. Given that PAS frequently involves the region near the internal cervical os in placenta previa [15, 16], transvaginal ultrasonography (TVUS) offers practical advantages for intraoperative use. However, no studies have evaluated intraoperative blood flow at the placental attachment site or its association with PAS. The clinical significance of such hemodynamic assessments remains unclear.

This study aimed to evaluate intraoperative temporal changes in blood flow within cervical hypervascularity using ultrasonography and to examine their association with PAS. We further assessed the predictive accuracy of blood flow duration for PAS and explored optimal cut‐off values to determine the clinical utility of this method in facilitating early intraoperative diagnosis and management of PAS.

## 2 Methods

### 2.1 Study Design

This retrospective observational study included patients diagnosed with placenta previa who delivered at Okayama University Hospital (Okayama, Japan) between January 2017 and June 2024. Clinical data were obtained from electronic medical records, conference documentation, operative notes, and surgical video recordings. The study was approved by the Ethics Committee of Okayama University Hospital (Approval No.: 2409‐047), and relevant information was disclosed to eligible patients via an opt‐out approach.

Among the 3080 deliveries that occurred at our institution during the study period, we included patients who met the following criteria: singleton pregnancy, age ≥ 18 years, diagnosis of placenta previa, delivery by cesarean section, and availability of intraoperative TVUS data for blood flow assessment. Medical records, conference materials, surgical reports, and intraoperative videos were reviewed for all eligible patients. Exclusion criteria were multiple pregnancies, cesarean deliveries performed entirely under general anesthesia from the outset of the procedure, absence of intraoperative TVUS blood flow recordings or documentation, stillbirths, marginal placenta previa, and low‐lying placenta.

### 2.2 Preoperative Risk Assessment for PAS in Placenta Previa

**FIGURE 1:**
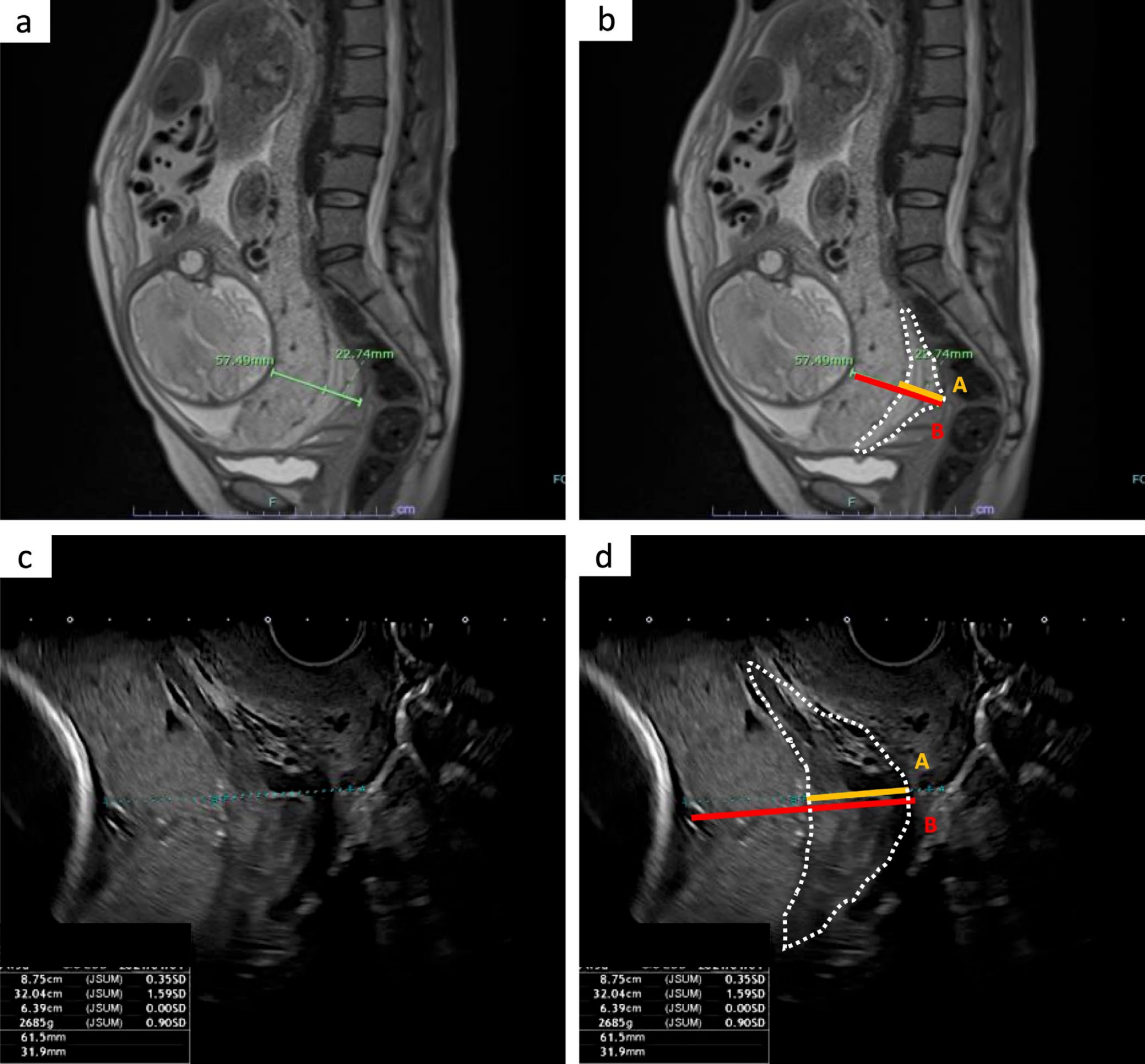
Imaging of cervical hypervascularity during pregnancy: TVUS and MRI findings. These images include sagittal T2‐weighted MRI scans (a and b) obtained at 33 weeks of gestation and transvaginal ultrasonographic images (c and d) acquired at 35 weeks in a patient with placenta previa. The region outlined by the white dashed line in panels (b) and (d) represents the cervical varicosity, which was defined based on its characteristic vascular morphology along the cervical canal. The positional relationship between the cervical varicosity, the placenta, and the serosal surface is indicated as A/B.

Placenta previa was defined as a condition in which the placenta completely or partially covered the internal cervical os [17]. In cases diagnosed with placenta previa via TVUS prior to 32 weeks of gestation during routine antenatal checkups, further detailed assessment of placental adherence was undertaken at approximately 33 weeks using both MRI and TVUS. MRI findings suggestive of abnormal placental adherence were interpreted by certified radiologists and included the presence of dark intraplacental bands on T2‐weighted images, heterogeneous placental signal intensity, indistinct borders between the placenta and myometrium, myometrial thinning or interruption, and focal uterine bulging [1, 18, 19, 20]. Additionally, based on the method proposed by Ishibashi et al. for predicting PAS using the ratio of cervical varicosity depth to placental thickness, sagittal T2‐weighted images including the internal cervical os were used [4, 5, 21]. To quantify cervical hypervascularity, we calculated the A/B ratio [4], where “A” represented the shortest distance from the most posterior cervical varicosity to the decidual surface of the placenta, and “B” represented the shortest distance from the same varicosity to the amniotic side of the placenta (Figure 1). In cases where the multidisciplinary team assessed a high risk of PAS and considered hysterectomy likely, preoperative preparations such as ureteric stent placement or temporary balloon occlusion of the internal iliac arteries were carried out immediately prior to surgery. In all such cases, 300–900 mL of autologous blood was prepared in advance.

### 2.3 Primary Outcome Measurement (Intraoperative Transvaginal Ultrasound Assessment of Cervical Blood Flow)

**FIGURE 2:**
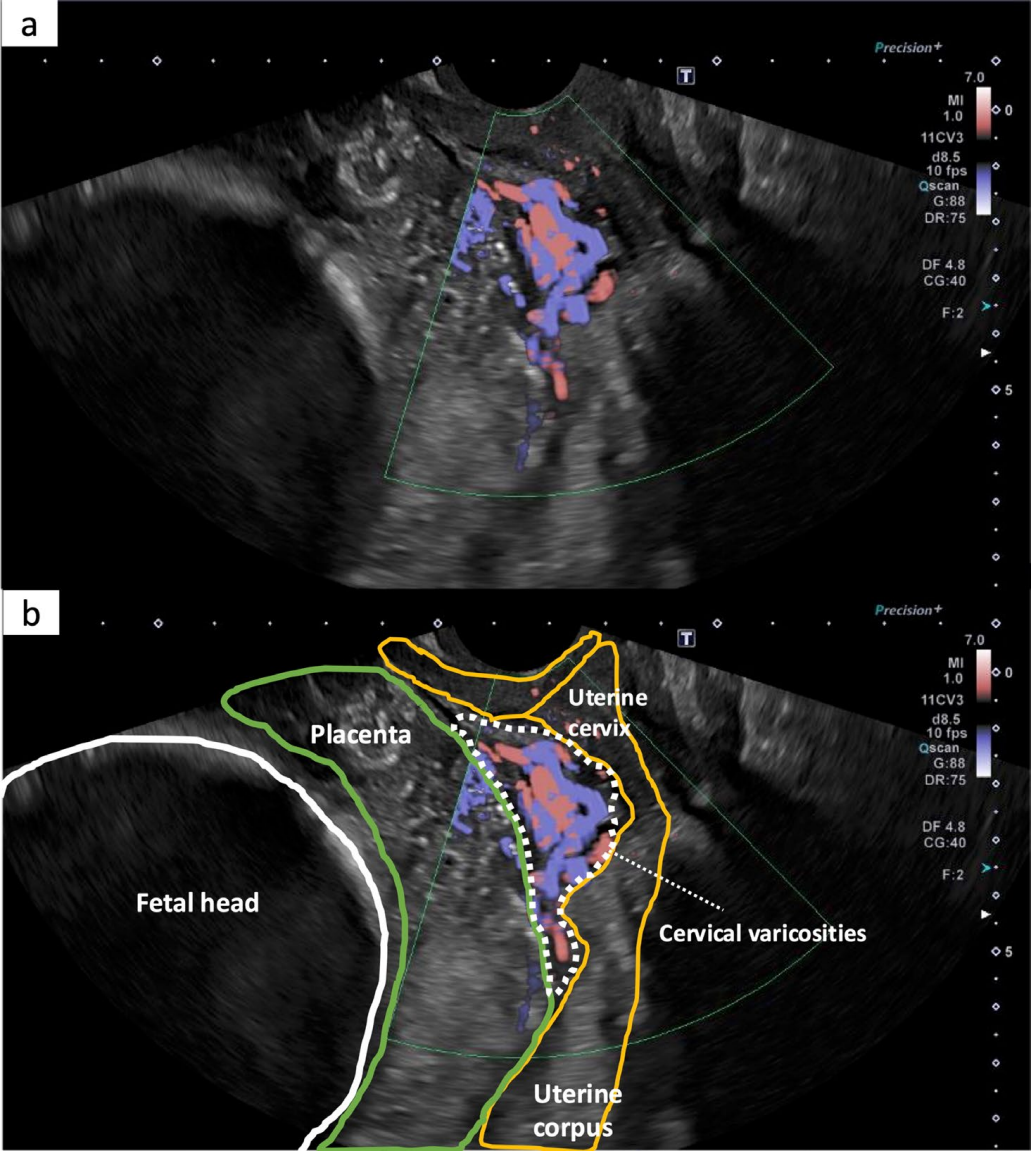
Real time color Doppler imaging of cervical hypervascularity at the beginning of cesarean section in a case of placenta previa. Panels (a) and (b) show transvaginal ultrasonographic images obtained at the start of surgery, prior to fetal delivery, captured within the same time frame. Blood flow within the cervical varicosity is assessed in the sagittal plane using color Doppler mode. Panel (b) includes color overlays to illustrate anatomical orientation.

The primary outcome of this study was the intraoperative evaluation of blood flow using TVUS. Patients were placed in the lithotomy position, with a dedicated ultrasound examiner positioned between the legs. A transvaginal probe (5–9 MHz; Canon Aplio i800, Tokyo, Japan) was used. In the sagittal plane on greyscale imaging, the target imaging plane was defined as the plane showing the cervical line extending from the external to the internal cervical os. Within this plane, color flow signals corresponding to cervical hypervascularity located between the placenta and myometrium were assessed (Figure 2). Color Doppler mode was employed to detect low‐velocity blood flow (2.0–12.0 cm/s), using the preoperative color flow signal as a reference.

Intraoperatively, continuous observation of cervical hypervascularity was conducted from fetal delivery until placental delivery. The duration of detectable blood flow, referred to as the blood flow persistence time, was defined as the interval from fetal delivery to the disappearance of the color flow signal (velocity ≤ 2.0 cm/s), based on the preoperative reference. A velocity threshold of ≤ 2.0 cm/s was selected because, at this level, color flow signals typically become undetectable in Color Doppler mode due to technical limitations in visualizing very low velocity flow. Thus, this value serves as a practical indicator of blood flow cessation in patients with cervical hypervascularity. The blood flow persistence time was recorded in minutes. Each case was retrospectively reviewed using operative video footage and surgical records. Two obstetricians specializing in perinatal medicine independently measured the blood flow persistence time. Interobserver agreement was assessed using the kappa coefficient. In cases of discrepancy between observers, the mean of the two measurements was used. For cases in which blood flow persisted beyond 40 min, the persistence time was uniformly recorded as 40 min. Two representative cases demonstrating the duration of blood flow within cervical hypervascularity, one diagnosed with PAS and one without, are presented as supplementary videos in Video S1.

### 2.4 Diagnosis of PAS

The presence or absence of PAS was assessed based on clinical and pathological findings, in accordance with the International Federation of Gynecology and Obstetrics (FIGO) classification system [22]. PAS was defined as grade 1 (abnormally adherent placenta; placenta adherenta or creta), grade 2 (abnormally invasive placenta; placenta increta), or grade 3 (abnormally invasive placenta; placenta percreta: 3a, 3b, and 3c). Specifically, grade 1 was diagnosed clinically during cesarean section when the following hallmarks were documented: failure of the placenta to detach spontaneously, the absence of a discernible retroplacental cleavage plane, and the necessity for forced manual removal. To address the potential for diagnostic subjectivity in grade 1 cases where the uterus was preserved, we performed a sub‐analysis by stratifying these cases into two groups: those with histopathological confirmation and those based on the aforementioned clinical criteria alone. Histopathological confirmation was defined as the identification of chorionic villi attached directly to the myometrium or the presence of myometrial fibers on the placental maternal surface.

### 2.5 Major Adverse Outcome

The major adverse outcome assessed in this study was peripartum hysterectomy. No maternal deaths related to placenta previa occurred during the study period.

### 2.6 Other Outcomes and Operative Procedure

Additional clinical data collected included maternal age at delivery, parity, mode of conception, pregnancy‐related complications, gestational age at delivery, medical and surgical history (including prior PAS, uterine surgeries, dilatation and curettage, and endometriosis), smoking history, additional interventions for hemostasis, and estimated intraoperative blood loss. At the research institution, the primary principle during surgery for suspected PAS was to avoid excessive traction on the placenta after fetal delivery. Uterine massage was performed while awaiting signs of spontaneous placental separation such as a placental bulge. When the placenta was extensive and hemostasis was stable, the myometrial incision was closed, and interval hysterectomy, including uterine artery embolization if necessary, was considered. In cases of partial placental adherence, manual removal or dissection using an energy device was performed after ensuring adequate preparation for potential hemorrhage. Bleeding from the placental bed was controlled with compression sutures or intrauterine balloon tamponade as appropriate.

### 2.7 Statistical Analysis

The required sample size was estimated through power analysis to evaluate diagnostic accuracy. Assuming an expected area under the curve (AUC) of 0.80, a null hypothesis AUC of 0.60, a significance level of 0.05, and a statistical power of 90%, the minimum number of required cases was calculated to be 33, based on an assumed 1:2 ratio of PAS‐positive to PAS‐negative cases.

Receiver operating characteristic (ROC) curve analysis was performed to assess the diagnostic accuracy of intraoperative cervical varicosity blood flow persistence time in predicting PAS in patients with placenta previa. Blood flow persistence times beginning at 6 min were assessed at 1‐min intervals to evaluate the sensitivity, specificity, and predictive values associated with each threshold. The optimal cut‐off value was determined using the Youden index, and the corresponding sensitivity, specificity, positive predictive value (PPV), and negative predictive value (NPV) were calculated. For the multivariable analysis, logistic regression was conducted using blood flow persistence time, history of cesarean section, mode of conception, and preoperative PAS prediction (MRI A/B ratio) as covariates. There were no missing data regarding patient demographic characteristics or primary outcome measures. However, a small number of cases had missing values for the MRI‐based preoperative PAS assessment. Given the non‐essential nature of these variables in the primary analysis and the relatively small proportion of missing data, no imputation was performed. Cases with missing MRI data were excluded from analyses involving MRI findings. The de‐identified analytic dataset used for all statistical analyses in this study is available as Data S1. All statistical analyses were conducted using SAS V.9.4 TS1M8 (SAS Institute, Cary, NC, USA), while sensitivity analyses and figure generation were performed using Python (v3.13.0).

### 2.8 Sensitivity Analyses

Two sensitivity analyses were conducted to evaluate the robustness of our findings. In the first analysis, cases of increta and percreta were excluded in order to assess diagnostic accuracy specifically for placenta accreta. ROC analysis was repeated using only accreta cases as the positive group. In the second analysis, the primary analysis was repeated after including cases that had initially been excluded due to general anesthesia or marginal placenta previa to examine the impact of these exclusion criteria on diagnostic performance.

## 3 Results

### 3.1 Study Population and Baseline Characteristics

**FIGURE 3:**
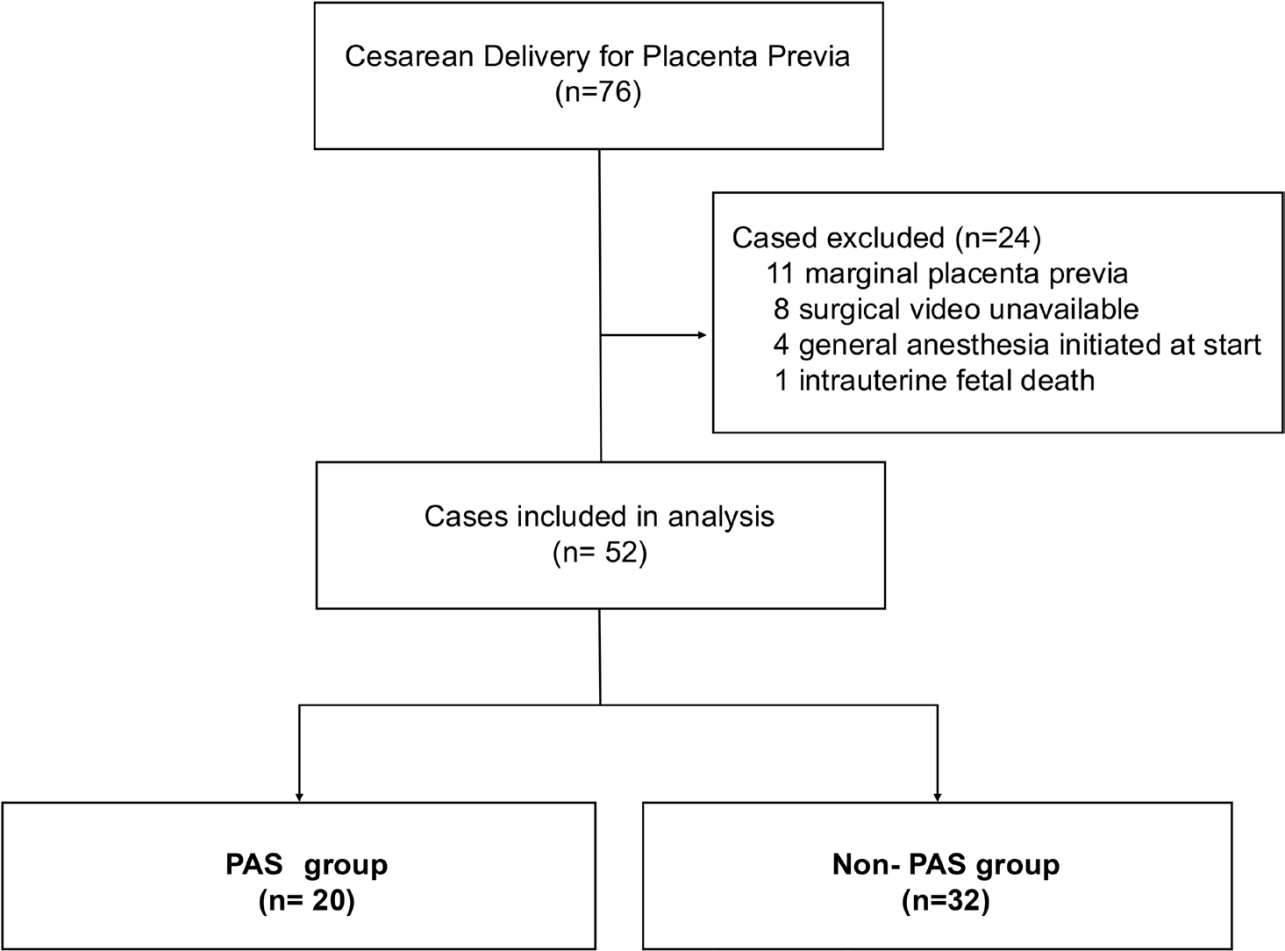
Flowchart of the study design.

Among the patients who underwent cesarean section for placenta previa, 76 cases were initially identified. After excluding 24 patients based on predefined criteria, 52 were included in the final analysis (Figure 3). Table 1 summarizes the maternal characteristics and clinical outcomes stratified by the presence or absence of PAS. The mean maternal age across all cases was 34.45 ± 4.97 years, and 24 of the 52 patients (46.2%) were nulliparous. PAS was diagnosed in 20 of the 52 patients (38.5%). Among those with PAS, a history of prior cesarean delivery was observed in 7 of 20 cases (35.0%), and a history of endometrial curettage was reported in 8 of 20 patients (40.0%). Regarding the mode of conception, in vitro fertilization was more frequent in the PAS group (8 of 20; 40.0%) than in the non‐PAS group (6 of 32; 18.8%), indicating a tendency toward higher prevalence in the PAS group. MRI A/B ratios were lower in the PAS group (0.26 ± 0.13) than in the non‐PAS group (0.38 ± 0.13). The mean duration of cervical varicosity blood flow, measured from fetal delivery, was 11.09 ± 11.64 min across all cases. In the PAS group, the duration was significantly longer, with a mean of 20.20 ± 14.31 min, compared to 5.69 ± 2.46 min in the non‐PAS group. The interobserver agreement for the assessment of blood flow duration was substantial, with a kappa coefficient of 0.67. Hysterectomy was performed in 7 of the 52 cases (13.5%), all of which occurred in the PAS group. Furthermore, we evaluated the relationship between PAS severity, clinical outcomes, and blood flow duration. Among the 20 PAS cases, 16 were classified as FIGO grade 1, 2 as grade 2, and 2 as grade 3 (Table 2). Peripartum hysterectomy was performed in 7 cases, consisting of 3 grade 1 cases, 2 grade 2 cases, and 2 grade 3 cases. In a sub‐analysis of grade 1 cases (*n* = 16), the median blood flow duration in the histopathologically confirmed group (*n* = 9) was 12.0 min (interquartile range [IQR], 10.0–36.0 min), whereas it was 8.0 min (IQR, 8.0–12.0 min) in the clinically diagnosed group (*n* = 7). The complete de‐identified dataset underlying the analyses presented in this section and subsequent sections is provided as Data S1.

**TABLE 1:** Comparison of clinical characteristics according to the presence of placenta accreta spectrum.

	PAS (+)		PAS (−)	
	*n* = 20		*n* = 32	
Age, mean years, SD	35.75	5.47	33.94	4.96
Parity, *n*, %				
Primiparous	8	40	16	50
Multiparous	12	60	16	50
Gynecological history, *n*, %				
Cesarean section	7	35	7	22
Placenta previa	2	10	1	3
Myomectomy	5	25	3	9
Endometrial curettage	8	40	7	22
Endometriosis	2	10	4	13
Conception, *n*, %				
Spontaneous conception	12	60	25	78
Intrauterine insemination	0	0	1	3
In vitro fertilization	8	40	6	19
Course of pregnancy, *n*, %				
Sentinel bleeding	12	60	18	56
Gestational diabetes mellitus	3	15	2	6
Pregnancy with uterine fibroids	5	25	5	16
Placental location, *n*, %				
Anteriorly dominant	9	45	7	22
Posteriorly dominant	11	55	25	78
Preoperative adhesion assessment				
MRI‐suspected adhesions^a^, *n*, %	14	74	9	31
MRI‐based A/B score of cervical hypervascularity^b^, mean score, SD	0.26	0.13	0.40	0.14
Intraoperative findings				
Bleeding, mL, SD	2523	2037	1594	711
Time to disappearance of blood flow within cervical hypervascularity, mean, SD	20.20	14.31	5.69	2.46
Placental delivery time, mean min, SD	23.04	22.11	8.52	5.41
Intraoperative interventions				
Manual placental removal, *n*, %	6	30	1	3.1
Bipolar vessel sealing device, *n*, %	6	30	5	15.6
Hemostatic suturing, *n*, %	6	30	12	37.5
Compression suture, *n*, %	2	10	4	12.5
Intrauterine balloon tamponade, *n*, %	10	50	12	37.5
Outcome				
Hysterectomy, *n*, %	7	35	0	0
Placenta accreta spectrum classification				
FIGO I	16	80	—	—
FIGO II	2	10	—	—
FIGO III	2	10	—	—

Abbreviations: FIGO, International Federation of Gynecology and Obstetrics; MRI, magnetic resonance image; PAS, placenta accreta spectrum; SD, standard deviation.

^a^ In the MRI examination, the presence of any one of the following four indicators suggestive of placenta accreta spectrum was considered to indicate a risk of placental adhesion: dark T2‐weighted intraplacental bands, heterogeneous signals in the placenta, myometrial thinning or interruption, and focal uterine bulging.

^b^ Cervical hypervascularity was evaluated using T2‐weighted sagittal images including the internal cervical os. The minimum distance from the most dorsal cervical hypervascularity to (A) the decidual (placental) side and (B) the amniotic side was measured and used for scoring.

**TABLE 2:** Clinical characteristics, intraoperative findings, and blood flow duration categorized by FIGO classification of placenta accreta spectrum.

	FIGO I *n* = 16		FIGO II *n* = 2		FIGO IIIa *n* = 2		FIGO IIIb	FIGO IIIc
Non‐hysterectomy cases (*n* = 13), %	13	81.2	0	0	0	0	0	0
Hysterectomy case (*n* = 7), %	3	18.8	2	100	2	100	0	0
Intraoperative findings								
Bleeding, mL, SD	2765	2123	640	410	2468	1741		
Time to disappearance of blood flow within cervical hypervascularity^a^, mean min, SD	15.3	11.3	> 40		> 40			

Abbreviations: FIGO, International Federation of Gynecology and Obstetrics; SD, standard deviation.

^a^ Blood flow duration values of “> 40 min” in grade 2 and grade 3 cases are right‐censored; the true disappearance time could not be measured because definitive intervention was initiated at that point. Because of the small sample sizes in grade 2 (*n* = 2) and grade 3 (*n* = 2), data are presented for descriptive purposes only and no inferential statistics were performed across grades.

### 3.2 Diagnostic Accuracy of Blood Flow Persistence Time for PAS

**FIGURE 4:**
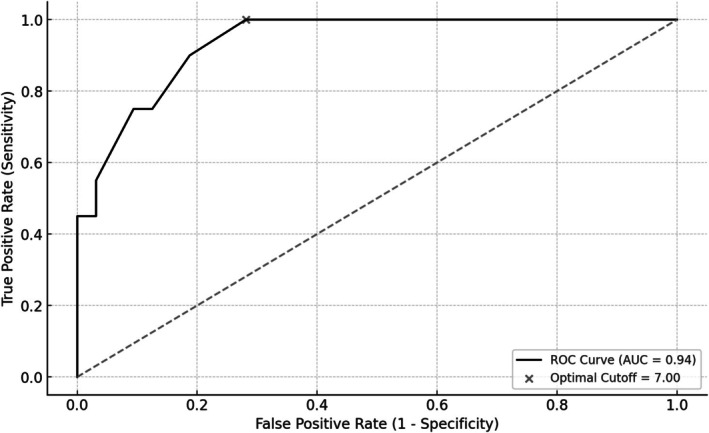
Receiver operating characteristic curve for the prediction of placenta accreta spectrum based on cervical varicosity blood flow persistence time. ROC curve illustrating the diagnostic performance of cervical varicosity blood flow persistence time in predicting the presence of placenta accreta spectrum.

**FIGURE 5:**
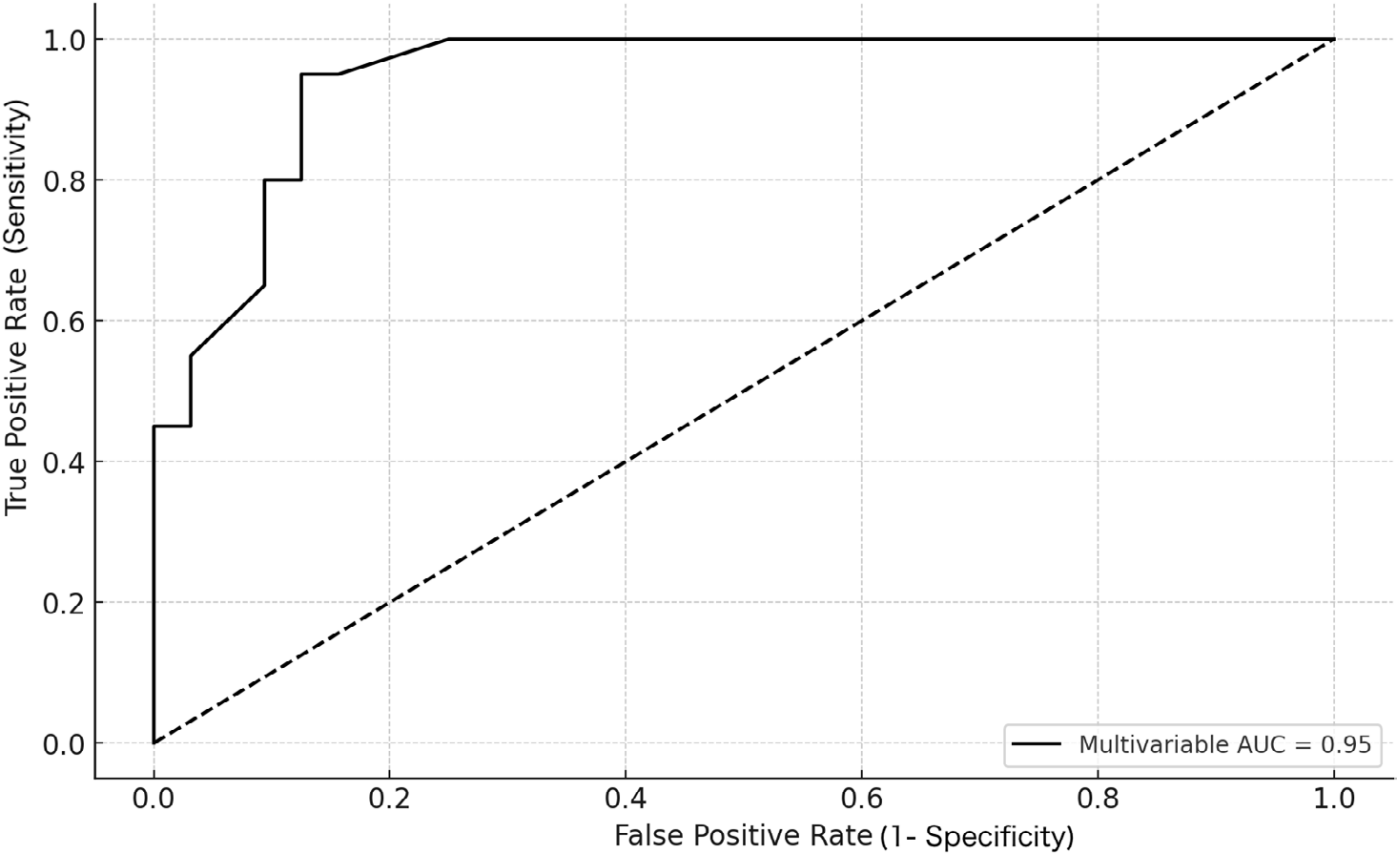
Multivariable ROC curve for the prediction of placenta accreta spectrum using blood flow persistence time and clinical covariates. Multivariate receiver operating characteristic curve assessing the diagnostic accuracy of a model predicting placenta accreta spectrum (PAS). The model incorporated cervical varicosity blood flow persistence time, history of cesarean section, mode of conception, and preoperative PAS prediction based on MRI A/B ratio as covariates.

Figure 4 presents the ROC curve assessing the diagnostic performance of cervical varicosity flow duration for the diagnosis of PAS. The AUC was 0.94, and the optimal cut‐off was 7 min. Table 3 summarizes the diagnostic performance metrics for cut‐off values ranging from 6 to 15 min. At the 7‐min threshold, sensitivity was 1.00, specificity was 0.72, the PPV was 0.69, and the NPV was 1.00. At 11 min, sensitivity decreased to 0.55, specificity increased to 0.97, PPV was 0.92, and NPV was 0.78. A blood flow persistence time of ≥ 11 min was highly predictive of PAS. Furthermore, a duration exceeding 14 min was observed in only a limited number of PAS cases but demonstrated a PPV of 1.00. The ROC curve in Figure 5 reflects the results of the multivariable analysis, which included cervical vascular flow duration alongside clinical and imaging predictors, demonstrating high diagnostic accuracy for PAS. The AUC following multivariate analysis was 0.95. The optimal cut‐off value for blood flow duration was 8 min, with a sensitivity of 0.95 and specificity of 0.91.

**TABLE 3:** Cut‐off levels of blood flow persistence time for the occurrence of placenta accreta spectrum.

Cutoff value for blood flow persistence time^a^ (min)	Sensitivity	Specificity	Positive predictive value	Negative predictive value
6	1.00	0.59	0.61	1.00
7	1.00	0.72	0.69	1.00
8	0.9	0.81	0.75	0.93
9	0.75	0.88	0.79	0.85
10	0.75	0.91	0.83	0.85
11	0.55	0.97	0.92	0.78
12	0.55	0.97	0.92	0.78
13	0.45	0.97	0.9	0.74
14	0.45	1.00	1.00	0.74
15	0.45	1.00	1.00	0.74

^a^ It is defined as the time from fetal delivery to the disappearance of the color flow signal based on the preoperative reference signal.

### 3.3 Predictive Value for Hysterectomy

The diagnostic performance of blood flow persistence time for predicting peripartum hysterectomy was evaluated, and the results are summarized in Table 4. At a cut‐off of 7 min, the sensitivity was 1.00, specificity was 0.51, the PPV was 0.24, and the NPV was 1.00. Specificity improved to 0.93 at a cut‐off of 13 min. When the threshold was set at ≥ 15 min, sensitivity remained at 1.00, specificity increased to 0.96, PPV was 0.78, and NPV remained at 1.00.

**TABLE 4:** Cutoff levels of blood flow persistence time for the occurrence of total abdominal hysterectomy.

Cutoff value for blood flow persistence time^a^ (min)	Sensitivity	Specificity	Positive predictive value	Negative predictive value
6	1.00	0.42	0.21	1.00
7	1.00	0.51	0.24	1.00
8	1.00	0.62	0.29	1.00
9	1.00	0.73	0.37	1.00
10	1.00	0.76	0.39	1.00
11	1.00	0.89	0.58	1.00
12	1.00	0.89	0.58	1.00
13	1.00	0.93	0.7	1.00
14	1.00	0.96	0.78	1.00
15	1.00	0.96	0.78	1.00
16	1.00	1.00	1.00	1.00

^a^ It is defined as the time from fetal delivery to the disappearance of the color flow signal based on the preoperative reference signal.

### 3.4 Sensitivity Analysis

For cases restricted to placenta accreta, the AUC was 0.92, with the optimal cut‐off point being 7 min; a flow duration exceeding 14 min yielded a PPV of 1.00 (Table S1, Figure S1). When cases involving general anesthesia and marginal placenta previa were included, the AUC remained high at 0.95, with an optimal cut‐off of 8 min, consistent with the main analysis (Table S2, Figure S2).

## 4 Discussion

This study evaluated the clinical utility of real time TVUS as an intraoperative diagnostic aid for PAS, focusing on the assessment of blood flow within cervical hypervascularity during cesarean section for placenta previa. A blood flow duration of less than 7 min was consistently associated with the absence of PAS, thereby supporting standard placental management with confidence. In contrast, a duration of ≥ 11 min demonstrated a strong association with PAS (PPV = 0.92), and all cases with flow lasting ≥ 14 min fulfilled the criteria for PAS (PPV = 1.00). These thresholds provide an objective and actionable parameter to guide intraoperative decision making. When blood flow persists beyond 7 min, a brief pause and team reassessment may facilitate timely preparation for escalated interventions, such as hysterectomy or massive transfusion, while minimizing the risk of hasty or forceful placental separation.

In the absence of PAS, placental separation occurs along the decidual interface, leading to the physiological collapse of maternal–fetal vascular shunts, including spiral artery flow. This results in the rapid cessation of blood flow within the uterine wall and cervical vascular layers [23, 24, 25]. In contrast, the physiological rationale for persistent flow in PAS aligns with established pathophysiological mechanisms. In PAS, failure of normal decidualization permits direct invasion of trophoblasts into the myometrium, serosa, or adjacent organs, resulting in the formation of abnormal arteriovenous communications between maternal and placental circulations [25, 26]. These pathological shunts often remain patent even after attempts at placental separation, allowing for sustained perfusion.

Particularly in the lower uterine segment and cervix, where collateral venous pathways such as the paracervical and parametrial plexuses are often well developed, blood flow may be maintained through both placental and extraplacental vascular supplies. Our intraoperative observations of persistent low‐velocity blood flow likely reflect these underlying structural and hemodynamic abnormalities in real time, and may serve as a non‐invasive physiological surrogate for pathological invasion in PAS. In this context, the persistence time of cervical hypervascularity represents a reproducible, physiological, and temporally responsive marker that can be assessed dynamically and non‐invasively. Unlike static imaging features such as placental lacunae or myometrial thinning [7, 18], flow duration permits continuous monitoring throughout the procedure, providing valuable intraoperative feedback as the surgical situation evolves. This is particularly advantageous when preoperative imaging findings are equivocal, inconsistent, or unavailable. Krapp et al. demonstrated that persistent color Doppler flow at the placental base could detect failed placental separation during the third stage of vaginal labor [25]. While conceptually similar, this study is fundamentally distinct in its clinical application. Specifically, by focusing exclusively on the cervical hypervascularity above the internal os rather than the entire placental bed, we target the area most prone to pathological vascular remodeling and severe adherence in placenta previa. Furthermore, our introduction of quantitative time cut‐offs such as 7 and 14 min transforms the binary observation of flow into an actionable surgical parameter. Pathophysiologically, the persistent flow within the cervical region reflects the sustained patency of abnormal arteriovenous communications through the paracervical venous plexuses, providing a more localized and clinically relevant indicator of placenta accreta spectrum severity in the context of cesarean delivery. Furthermore, our approach is conceptually distinct from the Sponge‐like echo, which is a static prenatal finding within the cervical myometrium. Unlike that morphological marker, we provide a dynamic intraoperative assessment specifically at the placenta‐myometrium interface to diagnose PAS.

Importantly, the present study highlights the inherent limitations of relying exclusively on preoperative imaging for the diagnosis of PAS. Conventional ultrasonographic indicators, such as the presence of multiple placental lacunae, discontinuity of the bladder wall, and attenuation or absence of the hypoechoic myometrial zone, have improved diagnostic accuracy [1, 3, 6, 7]. More recent MRI features, including dark intraplacental bands on T2‐weighted sequences, focal bulging of the uterine contour, and disruption of the uterine–placental interface [2, 7], have further enhanced diagnostic precision. However, these findings are not universally present in all affected cases. Moreover, interobserver variability and institutional differences in diagnostic experience may undermine the consistency of preoperative assessments. Such variability in risk stratification may contribute to suboptimal intraoperative management, including unnecessary uterine manipulation, delayed decisions regarding hysterectomy, or an increased risk of severe hemorrhage [12].

Several factors are known to contribute to adverse outcomes in cases of unexpected PAS, including delayed recognition, unplanned responses to hemorrhage, the use of inappropriate surgical techniques such as forceful placental removal, poor intrateam communication, and insufficient preparation [13, 27]. Intraoperative uncertainty often gives rise to confusion and panic, increasing the likelihood of maternal morbidity. An objective marker such as cervical blood flow duration could help bridge this gap by aligning team understanding, reducing cognitive bias, and supporting shared decision‐making in real time. Notably, real‐time intraoperative metrics are rare in obstetric surgery, and the introduction of a practical and reproducible marker represents a meaningful contribution to the field.

Based on these considerations, our institutional approach is centered on the principle of avoiding premature placental intervention. In our institutional practice, a core principle during intraoperative monitoring is the avoidance of premature umbilical cord traction or forceful manual placental removal while hemodynamically stable. This monitoring period is utilized for active management, such as performing uterine massage to assess for clinical signs of separation like a placental bulge, administering uterotonic agents, and initiating an intraoperative team huddle to reconfirm role assignments for potential hemorrhage. During this interval, we ensure logistical readiness, such as securing additional vascular access and blood products, while providing real‐time updates to patients to reduce anxiety. Our subsequent management is guided by the response to these interventions. If there are no signs of separation and bleeding remains minimal, we consider a staged surgical approach: closing the uterine incision followed by hysterectomy after uterine artery embolization. Conversely, if partial separation occurs but firm adherence persists, we utilize bipolar energy devices for sharp dissection after ensuring all preparations for escalated hemorrhage are complete. By integrating these active management steps with real‐time blood flow monitoring, we aim to standardize intraoperative decision‐making and enhance surgical safety.

This study is the first to evaluate blood flow duration as an intraoperative predictor of PAS. By quantifying a physiological parameter using the widely available TVUS, we propose a practical tool that may support intraoperative decision‐making and enhance surgical safety. However, it is important to acknowledge that the recommended intraoperative responses based on specific blood flow duration thresholds were developed within the context of our institution's patient population and surgical protocols. Therefore, these strategies may not be generalizable to all clinical settings. To support potential adaptation in other institutions, detailed decision‐making pathways and intraoperative management algorithms are provided in Table 5.

**TABLE 5:** Recommended intraoperative management steps based on cervical blood flow persistence time during cesarean section for placenta previa.

Flow duration range	Clinical interpretation	Recommended intraoperative actions	Notes
< 7 min	Low likelihood of PAS	Proceed with standard surgical procedure. Avoid unnecessary placental separation to ensure safety.	High negative predictive value (NPV).
7–11 min	Intermediate zone; moderate risk of PAS	Initiate intraoperative team huddle. Reassess surgical field. Prepare for hemostasis, blood transfusion, and consider potential need for TAH. Communicate with anesthesia and transfusion teams.	Surgical reassessment and team coordination needed.
11–14 min	High likelihood of PAS (PPV ≈ 92%)	Promptly notify anesthesiology and transfusion teams. Begin transfusion and prepare for surgical hemostasis. Reconfirm role assignments among surgical team.	Immediate action is critical.
≥ 14 min	PAS almost certain (PPV = 100%)	Prepare for TAH or staged surgery. Consider operator replacement and request experienced surgical backup. Ensure rapid team communication and coordination of intraoperative management.	All cases with ≥ 14 min were confirmed PAS.

Nonetheless, this study has several limitations. First, it was a retrospective, single‐center study, which may limit the generalizability of the findings. Second, although efforts were made to standardize the measurement of flow duration, the final classification relied on consensus between two experienced obstetricians, introducing a degree of subjectivity. Moreover, surgical outcomes such as hysterectomy were influenced not only by blood loss and placental adherence but also by surgeon experience, urgency of the case, and patient comorbidities. These confounding factors may affect the strength of the association between flow duration and clinical outcomes. Third, the direct causal relationship between persistent cervical blood flow and the pathophysiology of the PAS lesion remains a clinical hypothesis. Within the scope of this study, we could not provide direct evidence linking these regional hemodynamics to the degree of tissue invasion. Furthermore, several intraoperative confounding factors—including fluid resuscitation, systemic blood pressure management, individual variations in uterine contractility, and the specific site of the uterine incision—may have influenced the duration of cervical flow. Future prospective studies utilizing more objective physiological indicators under standardized protocols are necessary to control for these variables and further validate the underlying mechanisms. Moreover, the applicability of our method depends on the presence of visualizable cervical hypervascularity on intraoperative color Doppler. In cases with poorly developed or inconspicuous cervical vascularity, low‐velocity flow signals may fall below the detection threshold of TVUS, rendering blood flow duration unmeasurable or prone to false‐negative interpretation. We therefore cannot exclude the possibility that a subset of PAS cases lacking prominent hypervascularity may be missed when this approach is used in isolation; in such cases, integrated assessment combining intraoperative findings (placental bulge, cleavage plane) with preoperative imaging remains essential. Finally, while preoperative vascularity was assessed using the MRI A/B ratio [4], we lacked a specific intraoperative ultrasound index to quantify the degree of hypervascularity. The timing discrepancies of 1–2 min occasionally occurred when flow cessation coincided with uterine vibrations, as distinguishing low‐velocity signals from motion artifacts remains technically challenging.

Future prospective, multicenter studies are warranted to validate these findings and establish standardized thresholds for clinical use. In addition, efforts to automate and quantify flow duration using software or integrated Doppler imaging systems may further enhance reproducibility. Ultimately, the integration of cervical varicosity blood flow monitoring into obstetric surgical protocols may provide a simple, non‐invasive, and effective strategy to improve maternal safety and refine intraoperative diagnosis and management of PAS.

The intraoperative assessment of blood flow duration within cervical hypervascularity using TVUS offers a reproducible and physiologically meaningful marker suggestive of PAS in patients with placenta previa. The cessation of low‐velocity flow within 7 min following fetal delivery reliably excludes PAS, whereas persistent flow of 14 min or more is highly suggestive of its presence. Incorporating this objective parameter into intraoperative decision‐making may facilitate risk stratification, optimize surgical planning, and ultimately improve maternal safety during cesarean delivery for placenta previa.

## Author Contributions

**Kei Hayata:** conceptualization, methodology, writing – review and editing, resources. **Hikaru Ooba:** data curation, investigation, validation, formal analysis, supervision, visualization, writing – review and editing. **Tomohiro Mitoma:** conceptualization, methodology, data curation, investigation, writing – original draft, visualization, formal analysis, software. **Jota Maki:** conceptualization, methodology, supervision, project administration, resources, visualization, funding acquisition, writing – original draft, writing – review and editing. **Shujiro Sakata:** data curation, investigation, validation, writing – review and editing. **Ayano Suemori:** data curation, investigation, validation, visualization, writing – review and editing, resources.

## Funding

The authors have nothing to report.

## Disclosure

An earlier version of this article was presented at the 61st Annual Meeting of the Japan Society of Perinatal and Neonatal Medicine, which was held in Osaka, Japan, on 13–15 July 2025.

## Ethics Statement

This study was approved by the Ethics Committee of Okayama University Hospital (Approval No.: 2409‐047). In accordance with institutional policy, information regarding the study was disclosed to eligible patients via an opt‐out approach, and consent was obtained accordingly.

## Consent

The authors have nothing to report.

## Conflicts of Interest

The authors declare no conflicts of interest.

## Supporting information


**Table S1:** Sensitivity analysis of blood flow signal duration cutoff levels for diagnosing placenta accreta, excluding placenta increta and percreta cases.
**Table S2:** Sensitivity Analysis of Blood Flow Signal Cutoff Levels After Modification of Eligibility Criteria.
**Figure S1:** ROC Curve for the Diagnosis of Placenta Accreta After Excluding Increta and Percreta Cases.: Receiver operating characteristic (ROC) curve analysis repeated to assess the diagnostic accuracy of cervical varicosity blood flow persistence time for identifying placenta accreta, excluding cases of increta and percreta. The analysis was performed to isolate diagnostic performance for the most conservative form of placenta accreta spectrum.
**Figure S2:** Sensitivity Analysis Including Cases Initially Excluded Due to General Anesthesia or Marginal Placenta Previa.: ROC curve analysis repeated to evaluate the robustness of the diagnostic performance of cervical varicosity blood flow persistence time. This sensitivity analysis included cases that were initially excluded from the main analysis due to general anesthesia at the time of surgery or a diagnosis of marginal placenta previa. The aim was to assess the potential impact of these exclusion criteria on overall diagnostic accuracy.

**Video S1:** Intraoperative transvaginal ultrasonographic (sagittal view at the internal cervical os) temporal comparison between a non‐PAS case and a PAS case, demonstrating cervical hypervascularity located between the placenta and myometrium before and after fetal delivery, with measurement of the blood flow persistence time. In the PAS case, sequential images captured at 4, 10, and 20 min after fetal delivery show that the blood flow within the cervical hypervascularity remains essentially unchanged from the pre‐delivery state, in contrast to the rapid attenuation observed in the non‐PAS case.

**Data S1:** De‐identified patient‐level dataset (XLSX format) containing the variables used for all analyses in this study, including maternal age, parity, gestational age at delivery, prior cesarean history, placental location (anterior/posterior), preoperative ultrasonographic and MRI findings, intraoperative cervical varicosity blood flow persistence time (minutes), histopathological PAS classification per FIGO 2019, estimated blood loss, and occurrence of peripartum hysterectomy. All direct and indirect identifiers have been removed in compliance with the Ethics Committee approval (No. 2409–047, Okayama University Hospital).

## Data Availability

The de‐identified dataset supporting the findings of this study—including patient‐level clinical characteristics, intraoperative cervical blood flow persistence times, and PAS diagnostic outcomes used for the ROC and multivariable analyses—is provided as Supporting Information (Data S1) accompanying this article.
