# Supplementary material for: Intraoperative Transvaginal Ultrasonographic Evaluation for Placenta Accreta Spectrum in Placenta Previa: A Retrospective Observational Study
Source: J Obstet Gynaecol Res. 2026 Jun 4;52(6):e70353. doi: 10.1111/jog.70353 (PMC13238295; doi:10.1111/jog.70353)
Supplement: Supplementary file 1 — Table S1: Sensitivity analysis of blood flow signal duration cutoff levels for diagnosing placenta accreta, excluding placenta increta and percreta cases. Table S2: Sensitivity Analysis of Blood Flow Signal Cutoff Levels After Modification of Eligibility Criteria. Figure S1: ROC Curve for the Diagnosis of Placenta Accreta After Excluding Increta and Percreta Cases.: Receiver operating characteristic (ROC) curve analysis repeated to assess the diagnostic accuracy of cervical varicosity blood flow persistence time for identifying placenta accreta, excluding cases of increta and percreta. The analysis was performed to isolate diagnostic performance for the most conservative form of placenta accreta spectrum. Figure S2: Sensitivity Analysis Including Cases Initially Excluded Due to General Anesthesia or Marginal Placenta Previa.: ROC curve analysis repeated to evaluate the robustness of the diagnostic performance of cervical varicosity blood flow persistence time. This sensitivity analysis included cases that were initially excluded from the main analysis due to general anesthesia at the time of surgery or a diagnosis of marginal placenta previa. The aim was to assess the potential impact of these exclusion criteria on overall diagnostic accuracy. [file JOG-52-0-s002.docx]

| Table S1. Sensitivity analysis of blood flow signal duration cutoff levels for diagnosing placenta accreta, excluding placenta increta and percreta cases. | | | | |
| --- | --- | --- | --- | --- |
| Cutoff value for blood flow duration* (min) | Sensitivity | Specificity | Positive predictive value | Negative predictive value |
| 6 | 1.00 | 0.59 | 0.55 | 1.00 |
| 7 | 1.00 | 0.72 | 0.64 | 1.00 |
| 8 | 0.88 | 0.81 | 0.70 | 0.93 |
| 9 | 0.69 | 0.88 | 0.73 | 0.85 |
| 10 | 0.69 | 0.91 | 0.79 | 0.85 |
| 11 | 0.44 | 0.97 | 0.88 | 0.78 |
| 12 | 0.44 | 0.97 | 0.88 | 0.78 |
| 13 | 0.31 | 0.97 | 0.83 | 0.74 |
| 14 | 0.31 | 1.00 | 1.00 | 0.74 |
| 15 | 0.31 | 1.00 | 1.00 | 0.74 |
| *It is defined as the time from fetal delivery to the disappearance of the color flow signal based on the preoperative reference signal | | | | |

| Table S2. Sensitivity Analysis of Blood Flow Signal Cutoff Levels After Modification of Eligibility Criteria* | | | | |
| --- | --- | --- | --- | --- |
| Cut-off value for blood flow duration† (min) | Sensitivity | Specificity | Positive predictive value | Negative predictive value |
| 6 | 1.00 | 0.60 | 0.58 | 1.00 |
| 7 | 1.00 | 0.72 | 0.66 | 1.00 |
| 8 | 0.91 | 0.81 | 0.72 | 0.95 |
| 9 | 0.78 | 0.91 | 0.82 | 0.89 |
| 10 | 0.78 | 0.93 | 0.86 | 0.89 |
| 11 | 0.61 | 0.98 | 0.93 | 0.82 |
| 12 | 0.61 | 0.98 | 0.93 | 0.82 |
| 13 | 0.48 | 0.98 | 0.92 | 0.78 |
| 14 | 0.48 | 1.00 | 1.00 | 0.78 |
| 15 | 0.48 | 1.00 | 1.00 | 0.78 |
| *The main analysis was repeated after including cases that were initially excluded due to general anesthesia or marginal placenta previa, in order to examine the impact of these exclusion criteria on diagnostic performance. | | | | |
| † It is defined as the time from fetal delivery to the disappearance of the color flow signal based on the preoperative reference signal | | | | |


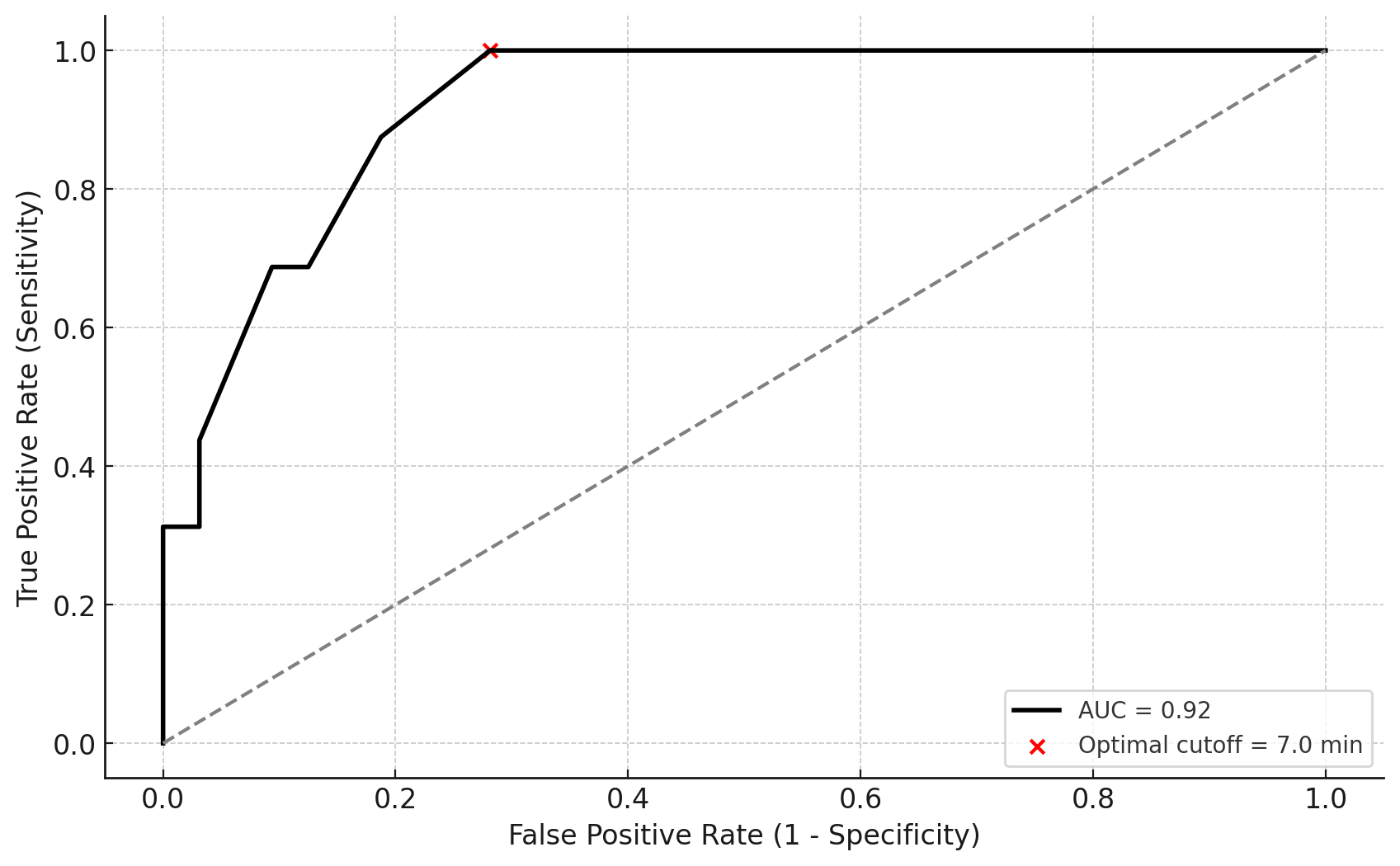


Figure S1. ROC Curve for the Diagnosis of Placenta Accreta After Excluding Increta and Percreta Cases

Receiver operating characteristic (ROC) curve analysis repeated to assess the diagnostic accuracy of cervical varicosity blood flow persistence time for identifying placenta accreta, excluding cases of increta and percreta. The analysis was performed to isolate diagnostic performance for the most conservative form of placenta accreta spectrum.


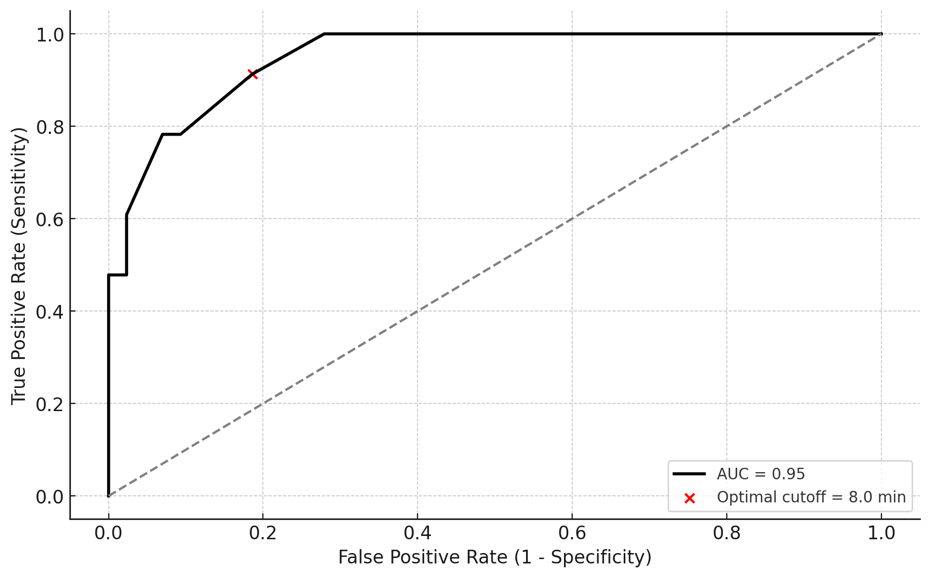


Figure S2. Sensitivity Analysis Including Cases Initially Excluded Due to General Anesthesia or Marginal Placenta Previa

ROC curve analysis repeated to evaluate the robustness of the diagnostic performance of cervical varicosity blood flow persistence time. This sensitivity analysis included cases that were initially excluded from the main analysis due to general anesthesia at the time of surgery or a diagnosis of marginal placenta previa. The aim was to assess the potential impact of these exclusion criteria on overall diagnostic accuracy.
